# Motor-Cortical Interaction in Gilles de la Tourette Syndrome

**DOI:** 10.1371/journal.pone.0027850

**Published:** 2012-01-04

**Authors:** Stephanie Franzkowiak, Bettina Pollok, Katja Biermann-Ruben, Martin Südmeyer, Jennifer Paszek, Götz Thomalla, Melanie Jonas, Michael Orth, Alexander Münchau, Alfons Schnitzler

**Affiliations:** 1 Medical Faculty, Institute of Clinical Neuroscience and Medical Psychology, University of Dusseldorf, Duesseldorf, Germany; 2 Department of Neurology, Medical Faculty, University of Dusseldorf, Dusseldorf, Germany; 3 Department of Neurology, University Medical Center Hamburg-Eppendorf, Hamburg, Germany; 4 Department of Neurology, University Hospital Ulm, Ulm, Germany; The University of Western Ontario, Canada

## Abstract

**Background:**

In Gilles de la Tourette syndrome (GTS) increased activation of the primary motor cortex (M1) before and during movement execution followed by increased inhibition after movement termination was reported. The present study aimed at investigating, whether this activation pattern is due to altered functional interaction between motor cortical areas.

**Methodology/Principal Findings:**

10 GTS-patients and 10 control subjects performed a self-paced finger movement task while neuromagnetic brain activity was recorded using Magnetoencephalography (MEG). Cerebro-cerebral coherence as a measure of functional interaction was calculated. During movement preparation and execution coherence between contralateral M1 and supplementary motor area (SMA) was significantly increased at beta-frequency in GTS-patients. After movement termination no significant differences between groups were evident.

**Conclusions/Significance:**

The present data suggest that increased M1 activation in GTS-patients might be due to increased functional interaction between SMA and M1 most likely reflecting a pathophysiological marker of GTS. The data extend previous findings of motor-cortical alterations in GTS by showing that local activation changes are associated with alterations of functional networks between premotor and primary motor areas. Interestingly enough, alterations were evident during preparation and execution of voluntary movements, which implies a general theme of increased motor-cortical interaction in GTS.

## Introduction

Gilles de la Tourette syndrome (GTS) is a common childhood onset neuropsychiatric disorder. It is characterized by multiple motor and phonic tics. Tics are brief movements that are misplaced in both context and time [1], [2]. Most patients report premonitory phenomena preceding tics described as an urge to move or other unpleasant sensations [3].

The pathophysiology of GTS is unclear. An abnormal processing within cortico-striato-thalamo-cortical-circuits associated with alterations of the dopaminergic neurotransmission has been suggested [4], [5]. Mink [6] postulated that a focal population of striatal neurons becomes abnormally active in GTS-patients leading to inhibition of globus pallidus pars interna and substantia nigra pars reticulata neurons increasing the excitability of motor-cortical areas. Along this line, alterations of the primary sensorimotor cortex and the SMA are assumed to play an important role in the pathophysiology of GTS [5], [7], [8], [9]. Accordingly, increased excitability of M1 at rest has been shown by means of transcranial magnetic stimulation (TMS) [10], [11], [12] which was related to tic-severity [13], [14]. Alterations of motor-cortical excitability in GTS during the execution of voluntary movements were investigated less intensively so far. During preparation and execution of voluntary movements a pattern of increased motor-cortical activation followed by increased inhibition was recently found using MEG [15]. Activation and inhibition of M1 were assessed by means of event-related desynchronization (ERD) and -synchronization (ERS). These measures reveal precise information about the temporal distribution of cortical activation patterns. Using functional magnetic resonance imaging (fMRI) two studies reported increased activation of primary sensorimotor and secondary motor cortices (primarily SMA) during the execution of a finger-tapping task [16], [17]. However, these analyses did not reveal information about a direct functional interaction between brain areas. As basal ganglia dysfunctions are assumed to play an important role in GTS the data described above imply that SMA and M1 might be abnormally driven by striatal neurons [6]. It is less well understood how the basal ganglia affect cortical activation patterns but, it is likely that functional interactions within a striato-thalamo-premotor-motor network are crucial for the observed excitability changes of the motor cortex of GTS patients. Accordingly, increased co-activation of SMA and M1 was observed preceding tics in GTS-patients but not preceding tic-imitation in healthy subjects [7]. Thus, functional interaction within a basal ganglia-thalamus-motor-cortical network is likely to be altered in patients with GTS.

Functional interaction between spatially distributed brain sites can be investigated by means of coherence between neural clusters. This approach requires methods with a temporal resolution in the range of milliseconds as revealed by electroencephalography or MEG. Due to its superior spatial resolution, MEG allows the detection of brain areas subserving task execution as well as the characterization of functional interaction within a given network [18], [19].

Since it has been argued that in GTS-patients abnormal activation of striatal neurons leads to disinhibition of a thalamo-cortical network, the present study aimed at investigating to what extent functional interaction within a thalamus-SMA-M1 motor control network is altered in GTS-patients. To this end, the functional network subserving preparation and execution of voluntary movements was characterized in GTS-patients as compared to healthy subjects. Since our previous data suggest increased activation of M1 during movement preparation and execution and increased inhibition after movement termination of voluntary movements [15], we here reanalyzed the same data in these time windows (i.e. time windows of ERD and ERS, respectively) in order to shed light on the functional interaction within a thalamo-motor-cortical network.

## Methods

### Ethics statement

All subjects gave their written informed consent prior to the study which has been approved by the Ethics Committee of the Hamburg Medical Association and which is in accordance with the Declaration of Helsinki.

### Patients and control subjects

In our previously published paper the data of 11 GTS-patients were analyzed with respect to ERD and ERS during a voluntary movement task. In the present study the data of ten patients (eight male; 35.7±3.1 years; mean ± standard error of mean (SEM)) were reanalyzed in terms of cerebro-cerebral coherence. Data from one patient was excluded from the analysis because of extensive movement artifacts during the MRT-scan. In the former study the MRI was not necessary since data were analyzed on the sensor level. Each patient was clinically assessed by an experienced neurologist or psychiatrist. Lifetime clinical information was systematically collected using a structured interview. GTS was diagnosed according to DSM-IV criteria. To measure the likelihood of having GTS we used the *Diagnostic Confidence Index* (DCI) [20]. Tic severity was rated using the *Yale Global Tic Severity Rating Scale* (YGTSS) [21]. Standardized video recordings were performed and data were scored using the *Modified Rush Videotape Rating Scale* (MRVS) [22]. Furthermore, tics per minute were counted during the video recording as described previously [14]. Patients fulfilling criteria of attention deficit hyperactivity disorder (ADHD), obsessive compulsive behaviour (OCB) or other psychiatric co-morbidities were excluded from the study. The diagnoses of ADHD and OCB were made according to DSM-IV criteria by using the *Adult ADHD Self-Report Scale* (ASRS-V1.1) [23] and the *Wender Utah Rating Scale* (WURS-k) [24]. All patients were off medical treatment for at least six months, respectively. Three patients did not receive medication at all.

Handedness was determined according to the *Edinburgh Handedness Inventory*. [25]. All except one patient were right handed. A summary of the clinical data is given in table 1. Additionally, 10 healthy volunteers matched with respect to age, gender and handedness served as control subjects (mean age 36±3 years).

**Table 1:** Clinical data.

						YGTSS					Tic-count: Tics/min							Medication	
											*relative tic number (%)*								
			Onset					motor	Total			Arm/	Hand/		Hip/				Drug free
Patient	Age	Sex	(age)	DCI	MRVS	total	tics	score	number	Face	Head	Shoulder	Finger	Body	Legs	complex	Vocal	Substance	since
P01	38	m	6	41	15	64	34	16	69	21,7	4,3	0	2,9	2,9	7,2	2,9	58,0	naïve	-
P02	28	m	6	63	14	49	29	18	62	54,8	22,6	4,8	1,6	0,0	4,8	8,1	3,2	T, P	>10 years
P03	28	f	8	36	6	9	9	9	35	60,0	0,0	11,4	17,1	0	5,7	5,7	0,0	T	>5 years
P04	42	f	3	76	13	42	22	17	63	38,1	22,2	3,2	1,6	1,6	7,9	1,6	23,8	D	>1 year
P05	54	m	13	57	15	49	19	11	68	10,3	22,1	19,1	4,4	14,7	10,3	7,4	11,8	H	>10 years
P06	38	m	11	45	6	22	12	9	15	80,0	13,3	0	0	0,0	0,0	0	6,7	T, P, C	>2 years
P07	25	m	5	67	7	29	19	13	41	51,2	14,6	12,2	12,2	0,0	7,3	2,4	0	naïve	-
P08	39	m	12	100	14	77	37	23	55	54,5	3,6	1,8	18,2	0,0	3,6	3,6	14,5	T, S	>6 month
P09	43	m	5	67	11	60	30	16	65	50,8	20,0	15,4	0	4,6	3,1	0	6,2	T, S	>2 years
P10	22	m	11	34	12	35	15	15	27	74,1	7,4	0	11,1	0	7,4	0	NA*	naïve	-
**Mean**	**35,7**		**8,0**	**58,6**	**11,3**	**43,6**	**22,6**	**14,7**	**50,0**	**49,6**	**13,0**	**6,8**	**6,9**	**2,4**	**5,7**	**3,2**	**13,8**		

*no sound in the videotape.

m = male, f = female; MRVS = total score of Modiefied Rush Videotape Rating Scale, Tic-count: tics per minute on video.

Total number = all tic counted; relative tic number (%) = all tics related to the respective muscle groups.

Medication: C = Clonidin, D = Dronabinol, H = Haloperidol, P = Pimozid, S = Sulpirid, T = Tiaprid.

### Paradigm

Patients and control subjects performed a self-paced finger movement task. They were instructed to execute voluntary brisk extensions of either the right index or the right middle finger in a randomized order at intervals of approximately 4 seconds. In total, at least 50 movements per finger were counted.

### Data collection

Subjects were comfortably seated in a magnetically shielded room while performing the task. The onset of finger movements was measured by two photoelectric barriers mounted on a pad. Neuromagnetic brain activity was recorded using a helmet shaped 122-channel whole-head neuromagnetometer (Neuromag™). Patients were video monitored during the measurement in order to determine tic episodes. Eye blinks were controlled by vertical and horizontal electrooculogram recordings (EOG) and bipolar electromyographic recordings (EMG) were used for further detection of tics. We monitored facial tics with unilateral electrodes at left frontalis muscle (lifting of eyebrows), left orbicularis oculi muscle (twinkle tic) and left orbicularis oris muscle (mouth tic). References were placed at the jaw. Shoulder tics were monitored with electrodes at bilateral trapezius muscle with reference at clavicles. MEG and EMG data were recorded with a bandpass filter of 0.03–330 Hz, digitized at a sampling rate of 1000 Hz, and stored digitally for off-line analyses.

The exact position of the head with respect to the MEG-sensor array was determined by measuring the magnetic signals of four coils fixated at the head of each subject. The coil positions were defined with respect to three anatomical landmarks - both preauricular points and the nasion - using a three-dimensional digitizer (Polhemus, VT). Individual high resolution T1-weighted MRIs were obtained for the alignment of MEG and MRI data.

### Data analyses

The number of tics and tic intervals were determined for each patient by visual inspection of EMG signals and video recordings. Epochs containing tics were excluded from further analyses. After applying a Hanning window, fast Fourier transform (FFT) was applied to all MEG signals using the Matlab FFT function (www.mathworks.com). FFT size was 1024 points. Windows overlapped with half the FFT size. Cross-spectral density was computed for all 122 channels and averaged across the whole measurement period. Alpha- (8–12 Hz) and beta-frequencies (13–24 Hz) were determined individually from FFT-spectra.

Brain areas subserving task execution, were detected using the oscillatory beamformer approach Dynamic Imaging of Coherent Sources (DICS) which employs a spatial filter algorithm and a realistic head model. DICS provides tomographic maps of oscillatory power and cerebro-cerebral coherence between brain sites in the entire brain (for details see [18]). Coherence is a normalized measure that quantifies dependencies in the frequency domain with values ranging from 0 (independent signals) to 1 (perfect linear relationship between two signals).

In a first step, the brain area with strongest oscillatory power within M1 in individual alpha- and beta frequency bands was determined, respectively (maximum FFT-peak ±2 Hz). This brain area was used as reference region for further coherence analyses between brain regions. The voxel showing strongest coherence towards the reference region was identified from local maxima of individual coherence maps and used for coherence analyses. In order to estimate a level of significance for cerebro-cerebral coupling, confidence limits were computed from surrogate data by randomly shuffling the original time courses, destroying all actual coherence. Only sources exceeding a 95% confidence level were taken into account for further analyses.

For visualization of mean group source localizations individual anatomical and functional data were normalized. Mean group data were displayed on a standard brain by means of SPM99 (Wellcome Department of Cognitive Neurology, Institute of Neurology, University College London, UK; www.fil.ion.ucl.ac.uk/spm). Please note that SPM was used for visualization only and does not provide any statistical comparisons between groups.

As a dysfunction of the basal ganglia is supposed to play a fundamental role in the GTS-pathophysiology we were interested in the functional interaction within a thalamo-motor-cortical network. Our analyses focused on coherence between the thalamus (as a relay station for basal ganglia input) and premotor and primary motor areas. Thus, the following connections were analyzed: thalamus - SMA, SMA - M1 bilaterally and M1 contralateral - M1 ipsilateral.

Spectral power and coupling strength between these sources were calculated at individual alpha- and beta-frequencies, respectively. Since our recent data indicate that motor cortical activation differs between the movement preparation and execution phase and the post-movement phase, power and coupling strength were calculated for the entire dataset and according to our previous data [15] for the time periods (i) of movement preparation and execution and (ii) the post-movement phase. Both time periods were defined with respect to individual ERD (corresponding to movement preparation and execution) and ERS (corresponding to the post-movement period) defined from our previous analyses. The time windows of ERD and ERS were determined based on the individual beta frequency modulation for each subject. While the ERD reflects a decrease of beta-activity below baseline level, the ERS reflects an increase of beta activity above the baseline. ERD usually starts before movement onset (reflecting movement preparation) and ends with movement termination [26]. ERD is followed by ERS most likely reflecting inhibition of neural circuits [27]. To determine the individual ERD/ERS time periods, the data were averaged with respect to movement onset for every subject using the analysis of temporal spectral evolution for each subject within the individual alpha and beta frequency band in a time window of 4 seconds prior to and after finger movement onset. As a pure resting baseline level was not evident, the interval between two succeeding finger movements was defined as baseline. ERD/ERS starting and ending time points were determined in each individual. For coherence analyses these individual time periods were used to ensure that the analysis selectively captures the two time periods of ERD and ERS.

In GTS-patients the movement preparation and execution phase on average lasted from 744 ms prior to movement onset to 53 ms after movement onset, in the control group from 749 ms to 13 ms after movement onset. The mean post-movement period was between 53 and 1101 ms after movement onset in GTS-patients and between 13 and 1068 ms in control subjects. Statistical analysis using Mann-Whitney-U Test revealed no significant differences between groups, neither for movement preparation/execution phase (p>0.05) nor for the post-movement phase (p>0.05).

Differences concerning local power and coupling strength between GTS-patients and control subjects were analyzed using Mann-Whitney-U-Test in SPSS 17.0 for Windows. For correlation analysis between electrophysiological data and clinical scores Spearman Rank Order correlation was used. Alpha adjustments for repeated test procedures were achieved with the sequentially rejective Bonferroni correction [28]. To quantify the direction of coupling between two oscillatory signals we calculated the directionality index (DI) that ranges between -1 and 1 [29]. While -1 and 1 corresponds to unidirectional coupling away and towards the reference region, respectively, 0 indicates bidirectional coupling between two signals.

## Results

The mean time interval between finger movements did not differ significantly between groups (GTS = 4170±760 ms; controls = 4364±1307 ms; p = 0.37). During the experiment (mean duration 378±31.7 seconds) tics occurred for 45±12.1 seconds (range 5–141 seconds) corresponding to 12% of the entire measurement time. These time intervals were excluded from data analyses. After artefact-rejection a total number of 104±11 epochs for the GTS patients and 102±21 epochs for the control group were included in the analyses. The amount of epochs did not differ significantly between groups (p>0.05).

### Source localization

**Figure 1:**
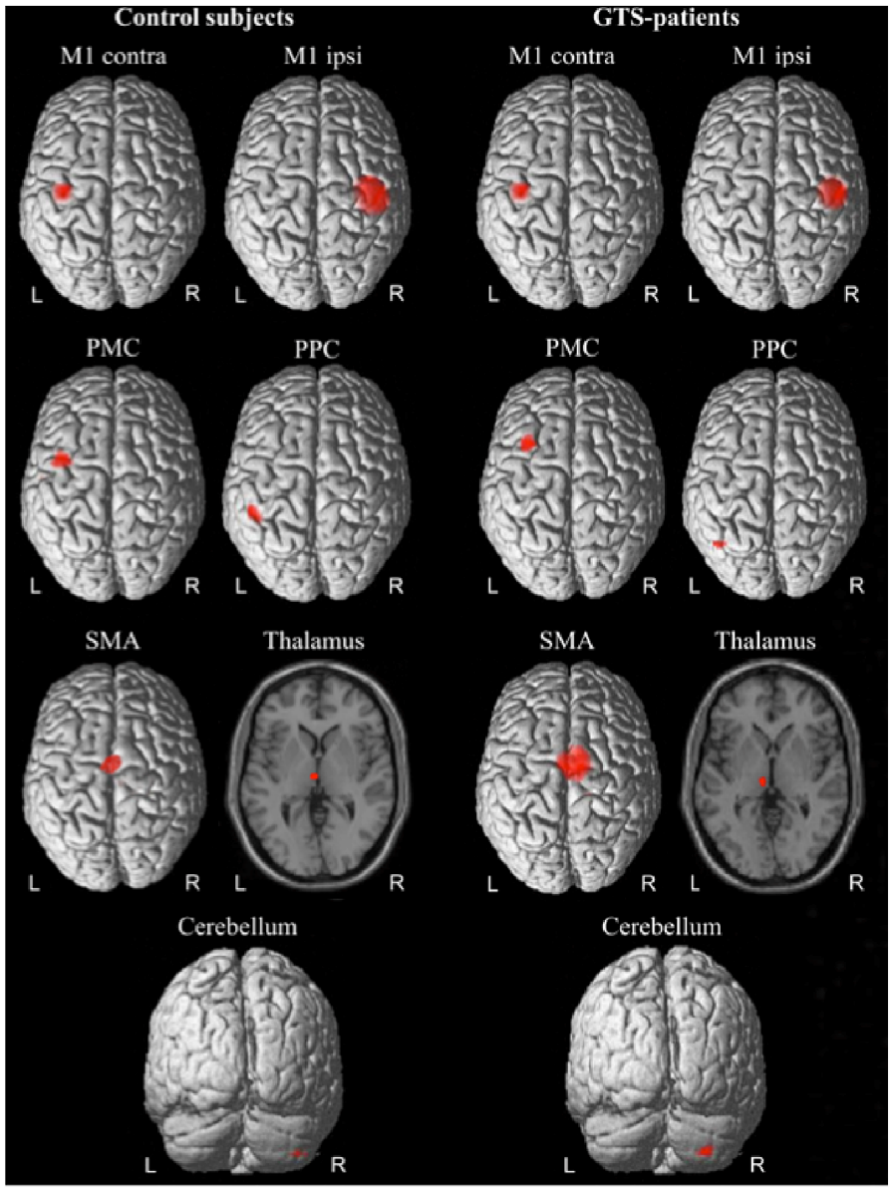
Mean localizations of all identified sources for control subjects (left) and GTS-patients (right). Please note that SPM99 has been used for visualization of mean source localizations only and does not provide any statistical comparison between groups.

In all patients and control subjects the voxel showing strongest power prior to the movement was localized within the hand area of M1 contralateral to the moving hand. Using this source as reference region we localized six coherent brain regions: ipsilateral M1 (10 controls, 10 patients), ipsilateral PMC (9 controls, 10 patients), ipsilateral posterior parietal cortex (PPC; 10 controls, 10 patients), SMA (9 controls, 9 patients), contralateral cerebellum (10 controls, 8 patients) and thalamus (10 controls, 10 patients). Figure 1 depicts mean source localizations of control subjects (left side) and patients (right side).

The appendant Talairach coordinates and Brodmann areas (BA) are summarized in table 2. Source localizations did not differ significantly between groups (p>0.05). Power as a measure of local activation was calculated for each source in each subject at alpha and beta frequencies, respectively. Again, statistical comparisons revealed no significant differences between patients and controls (p>0.05).

**Table 2:** Talairach coordinates.

		x-coordinate	y-coordinate	z-coordinate	
Source	Group	(mm)	(mm)	(mm)	BA
M1 contralateral	controls (n = 10)	−36	−22	50	4
	GTS (n = 10)	−38	−18	58	4
M1 ipsilateral	controls (n = 10)	44	−20	48	3
	GTS (n = 10)	42	−16	44	4
PMC contralateral	controls (n = 9)	−40	0	60	6
	GTS (n = 10)	−36	14	56	6
PPC contralateral	controls (n = 10)	−50	−36	58	40
	GTS (n = 10)	−44	−60	42	39
SMA	controls (n = 9)	10	−14	72	6
	GTS (n = 9)	6	−8	60	6
Thalamus contralateral	controls (n = 10)	0	−16	4	
	GTS (n = 10)	−6	−20	4	
Cerebellum ispilateral	controls (n = 10)	42	−72	−44	
	GTS (n = 8)	32	−86	−38	

### Coherence analyses

**Figure 2:**
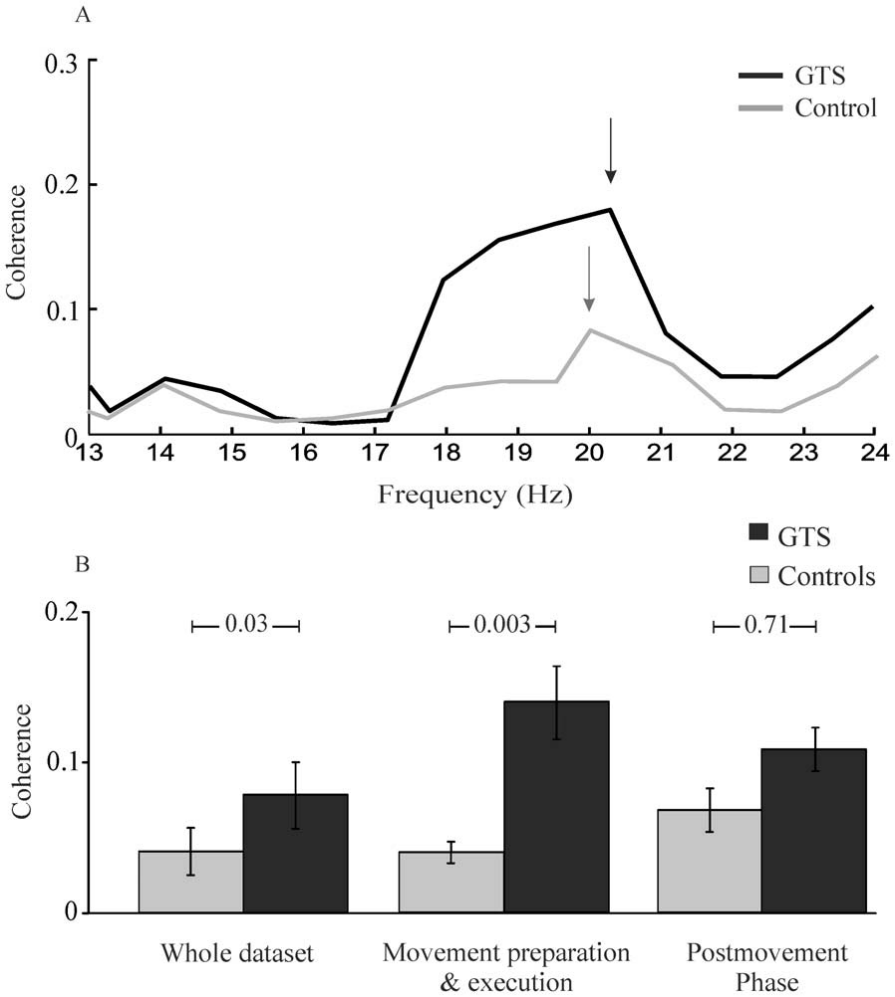
Coherence spectra between SMA and M1 of one representative GTS-patient (black) and one control subject (grey). The arrows mark the peak maxima (A). Mean M1-SMA coherence strength in GTS-patients (black) and healthy control subjects (grey; B). Error bars indicate SEM.

Maximal coherence peaks at alpha and beta frequencies were determined individually. In a first step coupling strength between all detected sources was calculated for the entire dataset. Figure 2A shows the coherence spectrum of an individual GTS-patient and a representative control subject. The group analyses revealed stronger coherence between left M1 (contralateral to the moving hand) and SMA at beta frequency in GTS-patients (GTS: 0.08±0.02; controls: 0.03±0.007; p_corrected_<0.05; figure 2B). On average, SMA-M1 coherence was maximal at 18.6±1.2 Hz in GTS-patients and at 19.8±0.8 Hz in control subjects. Peak frequencies did not differ significantly between groups (p>0.05). At alpha frequency no significant group differences were found (GTS: 0.06±0.03 controls: 0.04±0.01; p>0.05). Separating the dataset into time periods (i) of movement preparation/execution and (ii) a post-movement phase as described above resulted in significantly increased coherence between SMA and M1 contralateral during movement preparation/execution in GTS-patients (0.14±0.02) as compared to controls (0.04±0.007; p_corrected_<0.05). Analyses of the post-movement phase yielded no significant differences between groups (GTS: 0.1±0.03, controls: 0.07±0.01; p>0.05). To quantify the direction of coupling between SMA and MI the DI was calculated for GTS-patients (DI = 0.016±0.017) and control subjects (DI = −0.055±0.042). Since t-tests did not reveal significant difference to zero (p>0.05) values indicate bidirectional coupling.

Coherence analyses between other brain areas did not reveal significant differences between groups: thalamus - SMA (GTS: 0.07±0.01; controls: 0.09±0.03; >0.05), SMA - M1 ipsilateral (GTS: 0.14±0.03; controls: 0.14±0.03; p>0.05), M1 contralateral - M1 ipsilateral (GTS: 0.10±0.04; controls: 0.088±0,021; >0.05) in the whole and the temporally subdivided data set, respectively.

To assess if increased SMA-M1 coherence is related to tic severity, coherence strength during the movement preparation/execution phase was correlated with clinical parameters (i.e. YGTSS (Rho = 0.29), MRVS (Rho = 0.46), tics per minute (Rho = −0.017) and DCI (Rho = −0.033)). The analysis did not reveal significant results (p>0.19_uncorrected p-value_).

## Discussion

The present study – for the first time – directly investigates functional connectivity in GTS during the execution of voluntary movements. The results suggest increased functional coupling between SMA and contralateral M1 at beta-frequency. This was particularly evident during the movement preparation/execution phase - a time window in which increased M1 activation was recently found in GTS-patients [15]. At alpha-frequency no differences between groups were evident. Since beta oscillations are mainly generated in M1, the present results reflect alterations within the motor system [26], [30], [31].

It is well known that SMA is a key area for movement preparation in healthy subjects [32], [33]. Accordingly, functional coupling between SMA and M1 increases immediately before the execution of voluntary movements [34], [35], [36]. In the literature increased SMA activation has been reported in GTS [16], [17] during the performance of voluntary movements, a result that was not confirmed by the present data. Increased SMA activation in GTS has further been related to sensory urges [37], [38], [39]. Additionally, repetitive TMS at 1 Hz targeting the SMA showed improvement of tic severity as well as reduction of sensory urges in case series [38], [39]. In the present study patients were instructed not to suppress their tics and epochs containing tics were broadly cut out from the data. In sum, influences of sensory urges seem to be unlikely and that in turn might explain our present result concerning the lacking of differences between groups of SMA activation.

Increased activation of the sensorimotor cortex and SMA has been evidenced in previous fMRI-studies during the performance of voluntary tapping movements [16], [17]. Furthermore, using TMS, Heise et al. reported abnormal disinhibition of M1 during movement preparation that disappeared shortly before movement onset [40]. However, neither fMRI nor TMS data provide information about the functional interplay between brain sites. Our data extend and specify these results suggesting that increased M1 activation in GTS might be due to functional coupling with SMA. This hypothesis is particularly corroborated by the present finding that coupling was increased solely during movement preparation/execution but not in the post-movement phase. Since a recent study demonstrated a relationship between spontaneous oscillatory activity and motor-function related changes in M1 that is modulated by GABAergic mechanisms after application of a GABA-A modulator [41] it would be interesting to relate the present findings to patients' spontaneous beta oscillations. However, such analysis was not possible since we did not record resting brain activity in the present study. Moreover, all participating patients were OFF medication for at least six months, weakening the hypothesis that GABA-A agonists might have contributed to the present results.

The present finding can be interpreted along two lines. Firstly, increased SMA-M1 coherence might reflect an adaptive mechanism to facilitate the execution of voluntary movements. Secondly, it might represent a pathophysiological marker of GTS.

### Increased SMA-M1 coherence as adaptive mechanism

The hypothesis of adaptive mechanisms in GTS was made from several lines of research. For example, fMRI studies reported a correlation between the amount of structural changes of somatosensory cortex and tic severity [42], [43]. Using electroencephalography a cortical fronto-mesial network showing increased coherence during withholding of movements was observed in GTS indicating that the gain of inhibitory fronto-mesial networks is adaptively increased during suppression of voluntary movements and tics [8]. Several TMS studies reported reduced inhibition of M1 during rest, which was supposed to be linked to the release of involuntary movements. However, Orth et al. also reported reduced excitability of M1 during rest that correlates with tic severity. Hence, the better M1 excitability is reduced the better tics are controlled [14]. A recent TMS study investigated motor-cortical excitability during preparation of voluntary movements in GTS. The data indicate abnormal disinhibition of M1 during the early movement preparation phase. Shortly before movement onset inhibition increases and became similar to healthy controls. This might reflect a compensatory mechanism of top-down control from higher motor areas to override abnormal inputs from the basal ganglia to control motor cortical excitability [40] suggesting that GTS-patients can switch from a “tic state” associated with abnormal motor system excitability to a “voluntary movement state” paralleled by normalisation of motor cortex excitability.

### Increased SMA-M1 coherence as a pathophysiological marker of GTS

In GTS-patients pathologically increased activation within the basal ganglia is assumed to result in increased excitability of motor cortical areas, which has been related to the occurrence of tics [4], [6], [44]. Reduced basal ganglia volumes were observed in GTS-patients compared to healthy controls [45], [46]. There is convincing evidence that abnormalities of dopaminergic neurotransmission play an important role in the pathophysiology of GTS [47]. Therefore, the presumed aberrant striatal activity [48], [49] might be partly mediated by an overactive dopamine system by either an excess of dopamine or an increase in sensitivity to the neurotransmitter [50], [51], [52]. It is well known that SMA is a major target of projections from the basal ganglia [53]. Hence, one might argue that the observed coherence increase between SMA and contralateral M1 might be due to abnormal basal ganglia input causing over-activation of motor-cortical areas in GTS-patients. One possible mechanism leading to the exaggerated functional interaction might be based on abnormalities of dopaminergic functions within thalamocortical circuits. Recent studies using positron emission tomography revealed evidence that the dopaminergic dysregulation is a more generalized phenomenon evident also in the frontal cortex and the thalamus [54], [55]. Interestingly, the affected sites were localized - among others - within motor cortical areas [54]. Also postmortem studies point to a dopaminergic dysfunction in the frontal lobe and in the thalamus supporting the role of extrastriatal dopamine abnormalities contributing to the pathophysiology of GTS [56], [57]. Taken together, one could hypothesize that dopaminergic dysregulation might theoretically contribute to the increased SMA-M1 coherence reported in the present study. In the present study, coherence analyses did not yield significant differences of thalamus-SMA interaction between GTS-patients and controls. At first glance, this result argues against the hypothesis that increased motor cortical activation occurs due to a pathological drive from the basal ganglia. However, since MEG sensors are less sensitive to deeper brain areas this lack of evidence should be interpreted with caution. We had to deal with artifacts caused by tics. Artifact rejection yielded a reduced number of epochs and therefore led to reduced signal-to-noise ratio. Furthermore, the measured sample of 10 GTS-patients is rather small and might also explain the lacking difference.

Our data indicate that increased SMA-M1 coherence is also present during preparation/execution of voluntary movements suggesting a general theme of increased motor-cortical interaction in GTS. Since the SMA-M1 coherence strength is not correlated with tic severity we assume that increased coherence represents a general marker of GTS according to an all-or-nothing rule but does not reflect disease severity.

In a recent fMRI-study [7] SMA-M1 co-activation associated with tics as compared to healthy subjects mimicking such tics was investigated. This interaction was stronger prior to and after the performance of real tics. The present data extend these results (i) by directly showing differences of functional coupling between SMA and M1 particularly during movement preparation/execution which (ii) were evident while voluntary movements were performed. Therefore one might argue that increased SMA-M1 interaction is not likely to reflect solely tic-related brain activity or the presence of sensory urges. In fact, it might represent a pathophysiological marker that is evident in GTS *per se* during preparation/execution of any kind of movement regardless of tics. Our previous data [15] suggest increased M1 activation during movement preparation/execution observed in the same patient group indicating that SMA might influence M1 activation. Since coherence is a non-directed measurement we cannot clearly verify such top-down influence from SMA to M1. But, the present data did not reveal evidence for increased SMA activation. Thus, it is likely that SMA drives M1 yielding increased M1 activation while it is less likely that M1 drives SMA without affecting SMA activation. Therefore, although the analysis of the DI did not support a clear directionality from SMA to M1, it is less likely that the observed coherence pattern reflects directionality from M1 to SMA. Since our previous data suggest increased M1 activation during movement preparation/execution, we hypothesize that SMA drives M1 by pathologically increased functional interaction. We therefore favor the hypothesis that the observed pattern of SMA-M1 interaction in GTS might represent a pathophysiological marker instead of an adaptive mechanism.

One point that might argue against this interpretation is the fact that no differences of SMA-M1 interaction were observed during the post-movement phase. Our previous results revealed increased inhibition of the ipsilateral M1 after movement termination. Since inhibition was inversely correlated with tic severity, we interpreted this result in favour of a compensatory mechanism. The present results extent these findings by showing that such compensation is not mediated via SMA-M1 coherence. A limitation of the study is the lack of behavioural data. However, as the subjects performed a very simple task (i.e. finger lifts) it seems unlikely that differences of movement performance had contributed to the observed differences of SMA-M1 connectivity.

Another possible variable that might have influenced the present results is the requirement to select one of two fingers for the next response implying response switching. Recent studies have revealed evidence that GTS-patients exhibit greater levels of cognitive control during an oculomotor switching paradigm [58], [59]. This suggests an increased demand to monitor and control movements in GTS-patients. Additionally, it was reported that GTS-patients seem to be impaired in rapidly selecting or switching between different motor sets suggesting that patients exhibit deficits in the programming and planning of movement sequences without external visual cues [60]. Taken together, response switching requirements might serve as an alternative explanation for the present results.

Taken together the present and previous results, the data most likely point to a pathological alteration of functional interaction between premotor and primary motor areas presumably yielding increased M1 activity prior to and during the execution of voluntary movements.
